# Sampling depth of a diffuse reflectance spectroscopy probe for *in-vivo* physiological quantification of murine subcutaneous tumor allografts

**DOI:** 10.1117/1.JBO.23.8.085006

**Published:** 2018-08-27

**Authors:** Gage Greening, Ariel Mundo, Narasimhan Rajaram, Timothy J. Muldoon

**Affiliations:** University of Arkansas, Department of Biomedical Engineering, Fayetteville, Arkansas, United States

**Keywords:** diffuse reflectance spectroscopy, sampling depth, optical properties, absorption–scattering coefficient, hemoglobin, tissue oxygen saturation, murine subcutaneous tumor, allograft, colon, phantom

## Abstract

Diffuse reflectance spectroscopy (DRS) is a probe-based spectral biopsy technique used in cancer studies to quantify tissue reduced scattering ($\mu_{s}^{\prime }$) and absorption ($\mu_{a}$) coefficients and vary in source–detector separation (SDS) to fine-tune sampling depth. In subcutaneous murine tumor allografts or xenografts, a key design requirement is ensuring that the source light interrogates past the skin layer into the tumor without significantly sacrificing signal-to-noise ratio (target of ≥15  dB). To resolve this requirement, a DRS probe was designed with four SDSs (0.75, 2.00, 3.00, and 4.00 mm) to interrogate increasing tissue volumes between 450 and 900 nm. The goal was to quantify percent errors in extracting $\mu_{a}$ and $\mu_{s}^{\prime }$, and to quantify sampling depth into subcutaneous Balb/c-CT26 colon tumor allografts. Using an optical phantom-based experimental method, lookup-tables were constructed relating $\mu_{a},\mu_{s}^{\prime }$, diffuse reflectance, and sampling depth. Percent errors were <10% and 5% for extracting $\mu_{a}$ and $\mu_{s}^{\prime }$, respectively, for all SDSs. Sampling depth reached up to 1.6 mm at the first Q-band of hemoglobin at 542 nm, the key spectral region for quantifying tissue oxyhemoglobin concentration. This work shows that the DRS probe can accurately extract optical properties and the resultant physiological parameters such as total hemoglobin concentration and tissue oxygen saturation, from sufficient depth within subcutaneous Balb/c-CT26 colon tumor allografts. Methods described here can be generalized for other murine tumor models. Future work will explore the feasibility of the DRS in quantifying volumetric tumor perfusion in response to anticancer therapies.

## 1 Introduction

Diffuse reflectance spectroscopy (DRS) is a noninvasive, spectral biopsy technique that is used to indirectly estimate tissue optical properties and differentiate tissue types.^1^^,^^2^ The fundamental tissue optical properties are reduced scattering coefficient ($\mu_{s}^{\prime }$) and absorption coefficient ($\mu_{a}$).^3^ The $\mu_{s}^{\prime }$ morphologically depends on the size, density, and orientation of scattering particles in tissue, such as the cell membrane, cell nuclei, mitochondria, lysosomes, and collagen fibers, among others.^4^^,^^5^ In amelanotic tissues, the $\mu_{a}$ in the visible and near infrared spectral ranges functionally depends on the total hemoglobin concentration and tissue oxygen saturation.^3^ Changes in these fundamental optical properties have been shown to occur in neoplastic and cancerous tissue because of angiogenesis, degradation of stromal collagen, and altered morphology of epithelial cells.^5^^–^^7^ Therefore, DRS has shown promise for early cancer diagnostics, tracking tissue response to therapy, and in intraoperative surgical guidance.^1^^,^^2^^,^^8^^–^^12^

Spliethoff et al.^10^ used DRS to track changes in optical parameters over time in a mouse xenograft model of hereditary breast cancer in response to cisplatin chemotherapy. They showed that treated tumors had increased ${\mathrm{StO}}_{2}$ compared with nontreated tumors and concluded that DRS provided valuable functional tissue information that correlated well with tumor treatment response. This group also evaluated their fiber-optic needle-based DRS system on human lung cancer patients undergoing a diagnostic image-guided transthoracic needle biopsy procedure and concluded that spectroscopic guidance enabled more accurate needle positioning for lung biopsies.^9^ DRS has also been clinically applied to neurosurgery, in which Lin et al. performed DRS measurements on *in-vitro* brain tumors and developed a discrimination algorithm, primarily based on scattering from white matter, with a sensitivity and specificity of 96% and 93%, respectively.^11^ Recently, Hu et al. used DRS to measure tissue hypoxia in a subcutaneous mouse xenograft model of human pharynx squamous cell carcinoma treated with radiation and found that higher doses of radiation yielded a quicker increase in tumor oxygenation.^12^

DRS probes vary greatly in terms physical geometry and sampling depth depending on the tissue of interest. Physical geometry can differ in terms of probe length, probe tip diameter, number and type of integrated optical fibers, and degree of invasiveness. For example, most DRS probes contact the tissue surface and are considered noninvasive, but contact probes have limited sampling depth. Some groups have overcome this sampling depth limitation by creating minimally invasive, fiber-optic needle-based DRS systems;^10^ however, these systems sacrifice noninvasiveness and may induce bleeding at the tip of the needle, potentially affecting accuracy when quantifying total hemoglobin content. In noninvasive, contact-based DRS systems, sampling depth depends on source–detector separation (SDS), or the distance between the optical fibers delivering and collecting light. In general, as SDS increases, sampling depth increases due to the increased overall path length travel of the remitted photons at a cost of progressively decreasing signal-to-noise ratio (SNR).^13^^–^^15^ Thus, sampling depth can be fine-tuned to collect light primarily from specific tissue layers, such as epithelial, stromal, or subcutaneous tumor layers. Therefore, a relationship between raw diffuse reflectance, $\mu_{s}^{\prime },\mu_{a}$, and sampling depth must be established for each SDS channel.

Specifically, DRS can be used in subcutaneous murine tumors that are used for a variety of research purposes including investigating the effects of potential therapies.^16^ The central research question in this paper is: How can a DRS probe be optimally designed for evaluating tissue physiological parameters in subcutaneous murine tumors? At present, there have been no studies simultaneously quantifying wavelength- and SDS-dependent sampling depth in DRS probes with multiple channels to sample murine subcutaneous tumor allografts. The present study fills this knowledge gap by elaborating on methods to quantify wavelength-dependent sampling depth and demonstrating our capability to quantify physiologically relevant parameters such as total hemoglobin concentration (THC) and tissue oxygen saturation (${\mathrm{StO}}_{2}$), in subcutaneous murine tumors models. Experimental methods presented here are scalable for a variety of application-specific constraints, such as using small SDSs for endoscopically deployable probes within the subdiffuse regime, where the diffuse approximation is limited.^17^

A DRS probe was designed to interrogate subcutaneous murine tumors at increasing sampling depths and quantify the associated optical properties. The relationship between diffuse reflectance, $\mu_{s}^{\prime },\mu_{a}$, and SDS was experimentally established by measuring a set of tissue-simulating calibration phantoms to create lookup tables (LUTs). Then, the LUT was used as an inverse model to fit measured spectral data and extract optical properties.^18^^–^^20^ DRS data at each SDS represent a weighted average of physiological parameters collected at increasing depths. Therefore, a one-layer inverse experimental model was chosen to quantity volume-averaged, rather than layer-specific, physiological parameters without assuming precise thickness of overlying skin layers.^21^ The accuracy of the probe in extracting optical properties was determined using a second set of hemoglobin-based tissue-simulating phantoms. Following this, the relationship between sampling depth, $\mu_{s}^{\prime },\mu_{a}$, and SDS was experimentally established by detecting an embedded, highly absorbing, optical heterogeneity within tissue-simulating phantoms at incremental distances. Finally, the DRS technique was applied to a Balb/c murine allograft model of CT26 colon carcinoma as a model for subcutaneous mouse tumors. The $\mu_{s}^{\prime },\mu_{a}$, THC, ${\mathrm{StO}}_{2}$, and sampling depths were compared for normal and tumor tissues. The central hypothesis was that this probe would simultaneously sample the overlying epithelial skin layer as well as the subcutaneous Balb/c-CT26 tumor and accurately extract physiologically relevant optical parameters from each tissues.

## 2 Materials and Methods

### 2.1 Instrumentation

**Fig. 1:**
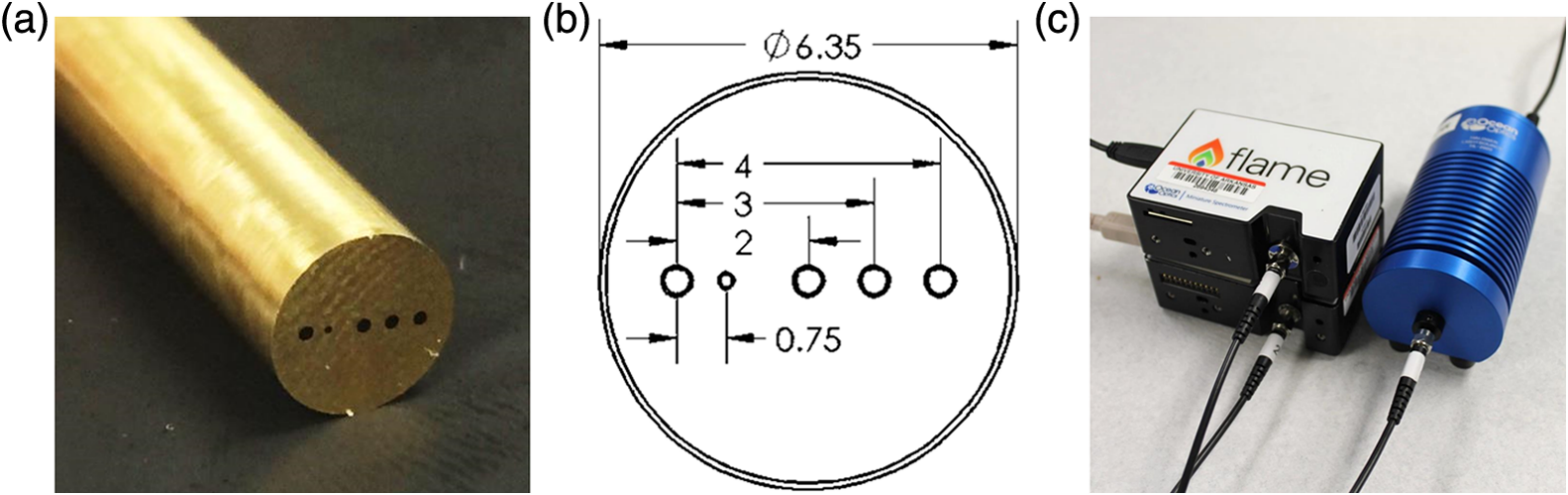
The DRS probe showing (a) distal optics, (b) dimensions of the optical fibers within the probe tip, and (c) proximal optics showing several legs of the DRS probe, spectrometers, and lamp.

The DRS probe (FiberTech Optica, Kitchener, Ontario, Canada) consists of a brass ferrule tip of 6.35 mm in diameter and 50 mm in length (Fig. 1). Five multimode optical fibers (NA=0.22±0.02, high-OH for wavelength range 190 to 1200 nm) are arranged in a slit line along the tip of the brass ferrule, with one fiber serving as the source fiber and the remaining four fibers serving as the detector fibers. SDSs are 0.75, 2.00, 3.00, and 4.00 mm. These optical fibers were included to sample into the subcutaneous murine tumor at increasing sampling depths. The source fiber as well as the 2.00-, 3.00-, and 4.00-SDS fibers (FiberTech Optica, SUV400/440PI) consist of a 400/440  μm±2% silica core/cladding with a 470  μm±5% polyimide jacket. The 0.75-mm SDS fiber (FiberTech Optica, SUV200/220PI) consists of a 200/220  μm±2% silica core/cladding with a 245  μm±5% polyimide jacket.

The total length of the DRS probe is 1.00 m. The distal (common) end of the probe is 0.67-m long, and fibers are secured within a 4.8-mm outer diameter black PVC-coating monocoil. The proximal (legs) end of the probe is 0.33 m in length, and each individual fiber is secured within a 3.0-mm outer diameter black PVC monocoil terminating in subminiature version A (SMA) connectors, reinforced with strain relief, to be attached to the lamp or spectrometers.

A 20W tungsten-halogen lamp (Ocean Optics, HL-2000-HP) provided broadband light (360 to 2400 nm) to the 400-μm core source fiber. One spectrometer (Ocean Optics, USB2000+VIS-NIR-ES) with a Sony ILX511B 2048-element linear silicon CCD array collected diffusely reflected light from the 0.75- and 2.00-mm SDSs. A second spectrometer (Ocean Optics, FLAME-S) with a Sony ILX511B 2048-element linear silicon CCD array collected diffusely reflected light from the 3.00- and 4.00-mm SDSs. The spectral resolution of the system [Eq. (1)] was calculated as

$$R_{\text{spectral}}(\mathrm{nm})=[\frac{{\text{Range}}_{\text{spectral}}(\mathrm{nm})}{{\text{Elements}}_{\text{pixel}}(\text{pixels})}]\times R_{\text{pixel}}(\text{pixels}),$$ (1)

where $R_{\text{spectral}}$ is the spectral resolution in nm, ${\text{Range}}_{\text{spectral}}$ is the spectral range that equaled 667 nm based on each spectrometer having a grating of 600 lines/nm, ${\text{Elements}}_{\text{pixel}}$ is the number of pixel elements that equaled 2048, and $R_{\text{pixel}}$ is the pixel resolution that equaled 6.5 pixels based on a 50-μm diameter laser cut slit within the round SMA connector (Ocean Optics, INTSMA-KIT). This resulted in a spectral resolution of 2.1 nm. No binning was performed.

### 2.2 Animal Model

The study was approved by the University of Arkansas Institutional Animal Care and Use Committee (IACUC #18060). CT26 (ATCC^®^, CRL-2638^™^), a murine colon carcinoma cell line derived from the Balb/c mouse strain, was maintained in Roswell Park Memorial Institute (RPMI)-1640 medium (ATCC^®^, 30-2001^™^) supplemented with 10% fetal bovine serum (ATCC^®^, 30-2020), 1% antibiotic antimycotic solution (Sigma-Aldrich, A5955-100ML), and 0.2% amphotericin B/gentamicin (Thermo Fisher Scientific, R015010). Third passage (P3) CT26 cells were used throughout the study.^22^

Ten female Balb/c mice were (strain: 000651, The Jackson Laboratory, ME) aged 9 weeks were housed in groups of three in three cages in the Small Animal Facility at the University of Arkansas. The facility was maintained at 23°C±1°C and 40% to 60% humidity on a 12:12 h light–dark cycle. Food (8640, Teklad) and water were provided *ad libitum*. All nine mice acclimated for 7 days after arrival prior to the study start. After 1 week of acclimation, the left flanks of the 10-week-old Balb/c mice were shaved, and Nair was applied for 1 min to locally remove hair. Then, $1\times 10^{5}$ CT26 cells in sterile saline were injected subcutaneously into the left flank.^23^^–^^25^ Tumor allografts grew until they reached a volume of $200\;{\mathrm{mm}}^{3}$, after which the tumor underwent DRS measurements.

### 2.3 Tumor Allograft Geometry

**Fig. 2:**
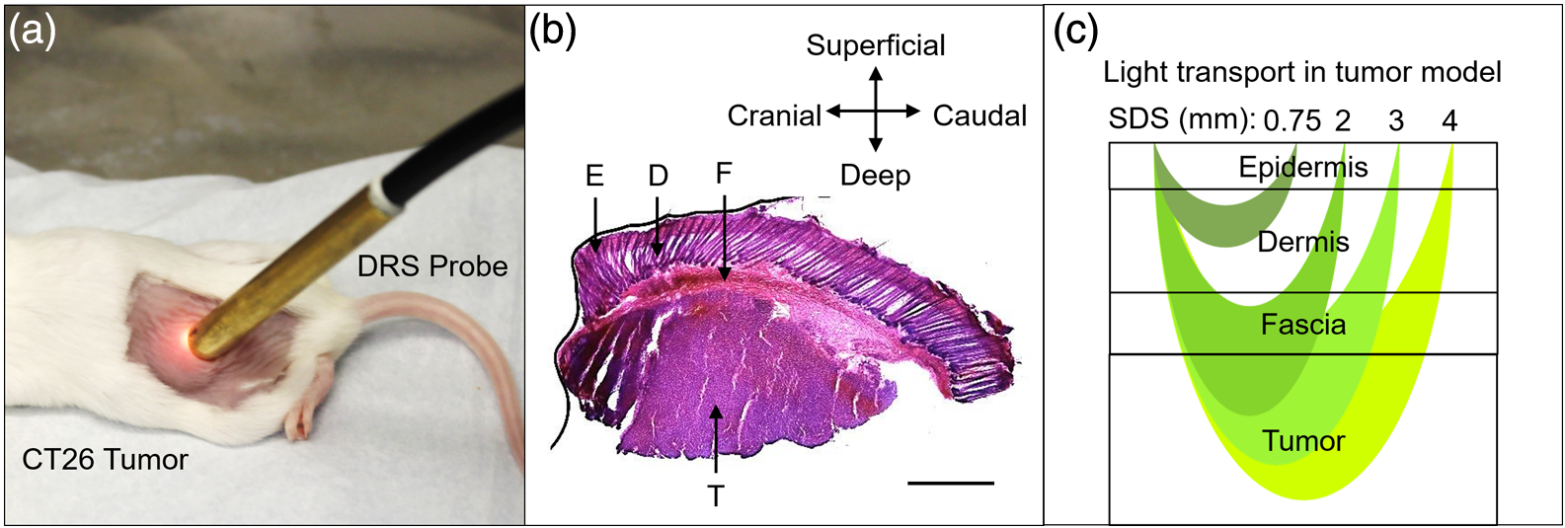
The subcutanous Balb/c-CT26 colon tumor allograft showing (a) the DRS probe in contact with the tumor, (b) an H&E-stained transverse section of tumor with overlying tissue layers (scale bar = 1 mm, E, epidermis; D, dermis; F, fascia; and T, tumor), and (c) a representation of light transport through the murine subcutaneous tumor allograft at each of the four SDSs (0.75, 2.00, 3.00, and 4.00).

After performing DRS measurements of Balb/c-CT26 tumor allografts at a volume of $200\pm 50\;{\mathrm{mm}}^{3}$, mice were euthanized via cervical dislocation under 4.0% isoflurane and 1 L/min oxygen. Tumors were dissected, placed in OCT and flash frozen in isopentane in liquid nitrogen, and stored at −80°C for up to 1 week. Tumors cut into 6-μm sections using a cryostat (Leica Biosystems CM1860) and stained with hematoxylin (VWR 100504-404) and eosin (VWR 10143-130) (H&E). H&E-stained tissue sections were imaged with a microscope (Nikon Eclipse Ci) with a 4X/0.25 NA objective and field of view (FOV) of 2.9×2.2  mm. Tumors often exceeded this FOV (i.e., a perfectly spherical tumor at a volume of $200\;{\mathrm{mm}}^{3}$ would have a diameter of ∼7.4  mm). Therefore, images were taken of the entire tumor cross section and stitched together using a commercial panoramic image stitching software (Microsoft, Image Composite Editor) (Fig. 2). Thickness of the epidermis, dermis/hypodermis, and fascia was calculated from H&E images calibrated to a 1951 USAF resolution test target (Thorlabs, R1DS1P). All nine CT26 tumors were measured to determine average and standard deviation. Calculating tissue thickness overlying the subcutaneous tumor was important to determine which layers were sampled by each SDS of the DRS probe (Fig. 2).

### 2.4 Optical Phantoms

To establish a relationship between optical properties, diffuse reflectance, and sampling depth in the LUT model, liquid calibration phantoms were generated with known $\mu_{s}^{\prime }$ and $\mu_{a}$. Calibration phantoms were constructed using distilled water as the solvent. The scattering agent was 1.00-μm-diameter polystyrene microspheres (07310-15, Polysciences) and the associated $\mu_{s}^{\prime }$ was calculated using Mie theory. The absorbing agent was teal India ink (11BY, Salis International). The $\mu_{a}$ was calculated by measuring a diluted solution of teal India ink in distilled water using a spectrophotometer (5102-00, PerkinElmer) and the Beer–Lambert Law.^18^^–^^20^^,^^26^

**Fig. 3:**
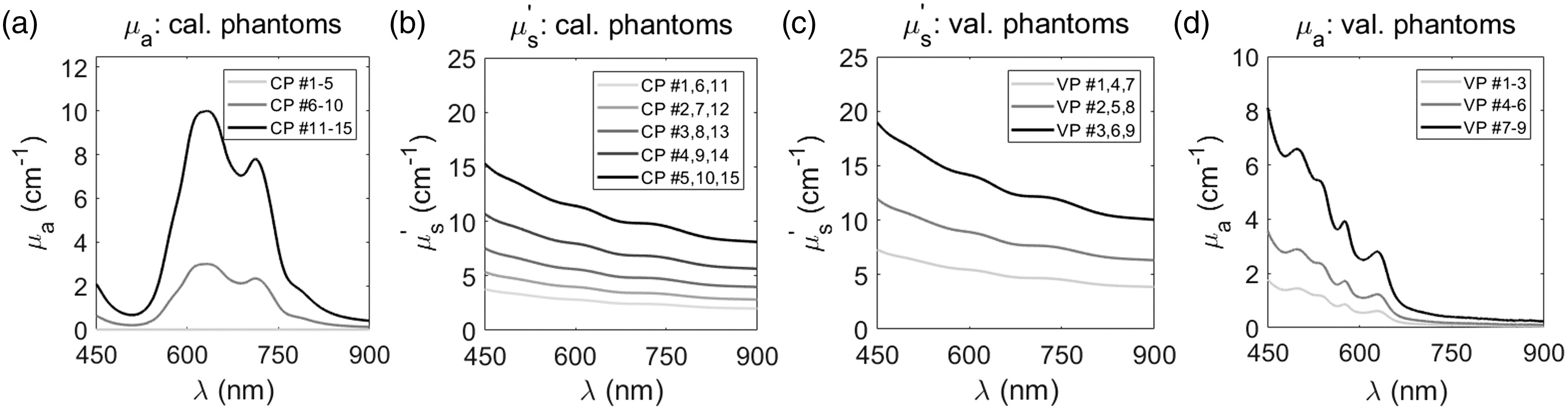
Calibration phantoms were made with distilled water, polystyrene microspheres, and teal India ink to span $\mu_{s}^{\prime }$ and $\mu_{a}$ ranges between (a) 2 to $15\;{\mathrm{cm}}^{-1}$ and (b) 0 to $10\;{\mathrm{cm}}^{-1}$, respectively, while validation phantoms were made with distilled water, polystyrene microspheres, and bovine hemoglobin to span $\mu_{s}^{\prime }$ and $\mu_{a}$ ranges between (c) 4 to $19\;{\mathrm{cm}}^{-1}$ and (d) 0 to $8\;{\mathrm{cm}}^{-1}$, respectively.

A 5×3 (15 total) set of calibration phantoms was created, corresponding to five scattering ranges and three absorbing ranges (Fig. 3). Five of the 15 phantoms were considered “scattering-only” and contained only polystyrene microspheres without India ink. Distilled water and polystyrene microspheres were mixed inside 7-mL scintillation vials (66022-300, VWR) to yield a $\mu_{s}^{\prime }$ of 2.7, 3.8, 5.4, 7.6, and $10.9\;{\mathrm{cm}}^{-1}$ at a reference of 630 nm to span a $\mu_{s}^{\prime }$ range of 2 to $15\;{\mathrm{cm}}^{-1}$ from 450 to 900 nm. The remaining 10 calibration phantoms contained both polystyrene spheres and teal India ink. Five of the 12 phantoms had a peak $\mu_{a}$ of 3.0 at $632\;{\mathrm{cm}}^{-1}$ and the final five phantoms had a peak $\mu_{a}$ of 10 at $632\;{\mathrm{cm}}^{-1}$. Thus, calibration phantoms spanned a $\mu_{s}^{\prime }$ range of 2 to $15\;{\mathrm{cm}}^{-1}$ and a $\mu_{a}$ range of 0 to $10\;{\mathrm{cm}}^{-1}$ from 450 to 900 nm. These ranges span the optical property range of interest for subcutaneous Balb/c-CT26 tumor allografts.^10^^,^^27^^,^^28^

To validate the relationship between optical properties and diffuse reflectance in the LUT model, liquid validation phantoms were generated with known $\mu_{s}^{\prime }$ and $\mu_{a}$, respectively. Using these validation phantoms, accuracy of the LUT model could be established by comparing known $\mu_{s}^{\prime }$ and $\mu_{a}$ (expected values) to the $\mu_{s}^{\prime }$ and $\mu_{a}$, respectively, generated by the LUT model (experimental). Validation phantoms were constructed similar to calibration phantoms but used bovine hemoglobin (H2625, Sigma-Aldrich) as the absorbing agent. Bovine hemoglobin was used to better simulate biological tissue absorption.

A 3×3 (9 total) set of validation phantoms was created, corresponding to three scattering ranges and three absorbing ranges (Fig. 3). Polystyrene microspheres were added such that phantoms yielded a $\mu_{s}^{\prime }$ of 5.2, 8.5, and $13.5\;{\mathrm{cm}}^{-1}$ at a reference of 630 nm to span a $\mu_{s}^{\prime }$ range of 4 to $19\;{\mathrm{cm}}^{-1}$ from 450 to 900 nm. Bovine hemoglobin was added such that phantoms yielded a $\mu_{a}$ of 0 to $1.8\;{\mathrm{cm}}^{-1}$, 0 to $3.6\;{\mathrm{cm}}^{-1}$, and 0 to $8.1\;{\mathrm{cm}}^{-1}$, respectively.

### 2.5 Lookup Tables for Diffuse Reflectance

The DRS probe was placed in each liquid calibration phantom, so it was completely submerged at 2 cm from the bottom of the 7-mL scintillation vial. Broadband DRS data (450 to 900 nm) were recorded at each SDS (0.75, 2.00, 3.00, and 4.00 mm) with integration times of 100, 200, 300, and 400 ms, respectively, to yield a SNR of at least 15 dB. Five spectra were averaged for all measurements. Spectra were converted to absolute diffuse reflectance values^21^ by calibrating with a Spectralon^®^ 20% diffuse reflectance standard (SRS-20-010, Labsphere), which accounts for the spectral shape and daily intensity fluctuations of the halogen lamp. Diffuse reflectance calibration [Eq. (2)] was calculated as

$$R(\lambda )=\frac{I_{\text{sample}}(\lambda )-I_{\text{background}}(\lambda )}{[I_{\mathrm{std}}(\lambda )-I_{\text{background}}(\lambda )]\times 100/R_{\mathrm{std}}},$$ (2)

where R(λ) is the absolute diffuse reflectance, $I_{\text{sample}}(\lambda )$ is the intensity of the raw, uncorrected spectra from phantoms or tissue, $I_{\text{background}}(\lambda )$ is the inherent background noise (spectra collected without excitation from the light source), $I_{\mathrm{std}}(\lambda )$ is the spectral intensity of the Spectralon^®^ 20% diffuse reflectance standard, and $100/R_{\mathrm{std}}$ accounts for the reflectance level (20%) of the Spectralon® diffuse reflectance standard, respectively. All intensity measurements per SDS were acquired with equal integration time.

**Fig. 4:**
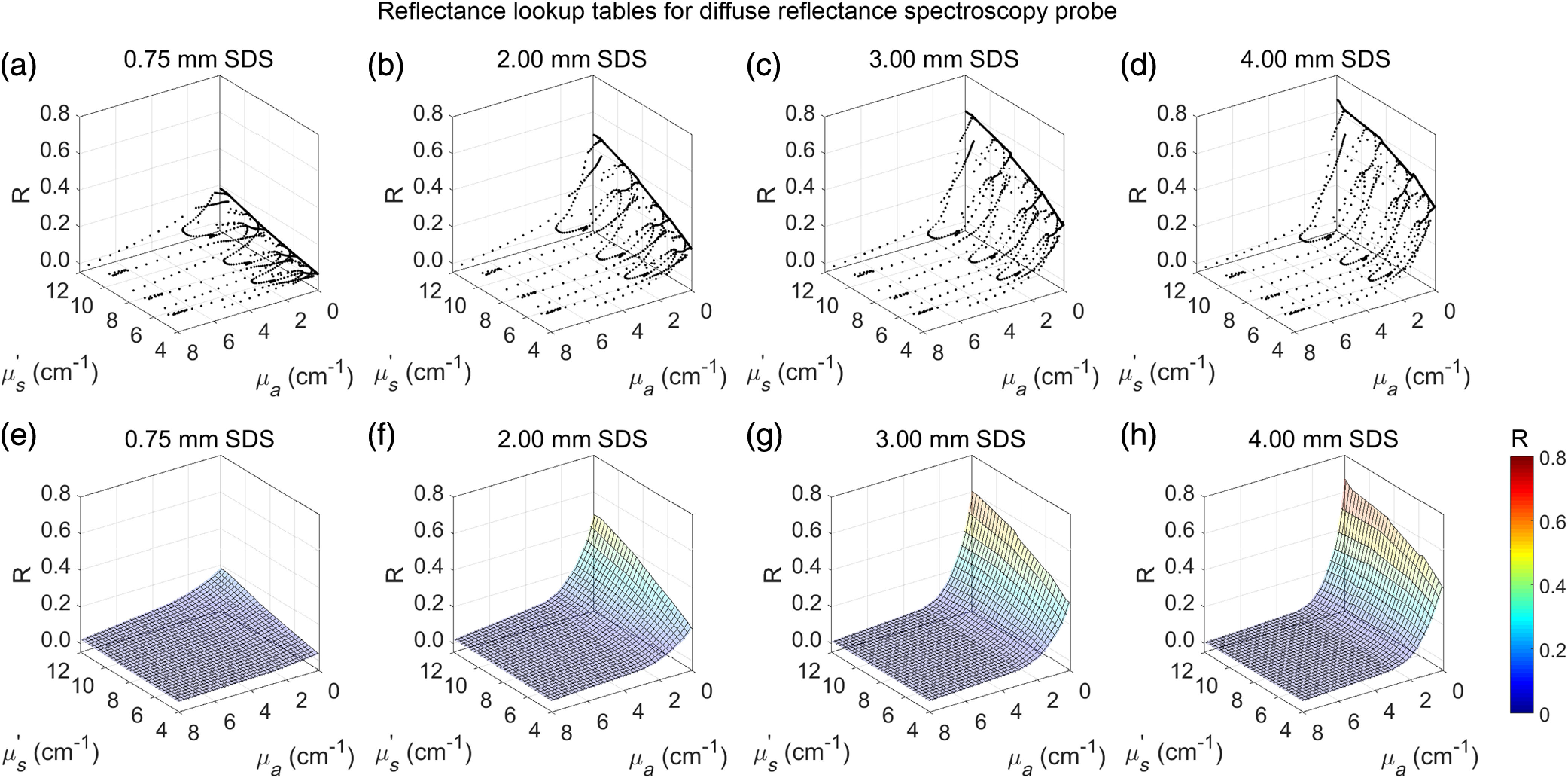
(a–d) LUTs were created for each SDS (0.75, 2.00, 3.00, and 4.00 mm) using diffuse reflectance spectra from calibration phantoms that were then (e–h) interpolated to create a continuous mesh for $\mu_{s}^{\prime }$ values between 4 and $12\;{\mathrm{cm}}^{-1}$ and $\mu_{a}$ values between 0 and $8\;{\mathrm{cm}}^{-1}$.

LUTs were generated for each SDS by plotting absolute diffuse reflectance (R) against $\mu_{s}^{\prime }$ and $\mu_{a}$ and then interpolating between raw data points to create a smooth mesh for $\mu_{s}^{\prime }$ between 4 and $12\;{\mathrm{cm}}^{-1}$, and $\mu_{a}$ between 0 and $-8\;{\mathrm{cm}}^{-1}$ (Fig. 4). This optical property range accounts for all expected $\mu_{s}^{\prime }$ and $\mu_{a}$ in murine tissue in the wavelength range of interest (450 to 900 nm).^27^

### 2.6 Validation of Lookup-Table Inverse Model

Once LUTs were constructed (Fig. 4), the accuracy of the LUTs needed to be quantified. In other words, for a single spectrum, how closely do the experimental optical properties (determined by the LUT model) match the expected optical properties?

The DRS probe was placed in each liquid bovine Hb-based validation phantom, so it was completely submerged 2 cm from the bottom of the 7-mL scintillation vial. Broadband DRS data (450 to 900 nm) were recorded at each SDS (0.75, 2.00, 3.00, and 4.00 mm) with integration times of 100, 200, 300, and 400 ms, respectively, to yield an SNR of at least 15 dB. Five spectra were averaged for all measurements. Spectra were converted to absolute diffuse reflectance values by calibrating with a Spectralon^®^ 20% diffuse reflectance standard and background noise subtraction as previously described.

Experimental $\mu_{s}^{\prime }$ and $\mu_{a}$ were calculated using the damped least-squares nonlinear fitting method, appropriate for least squares curve fitting. This method will be, henceforth, referred to as the LUT inverse model fit and was based on the constraining equation for $\mu_{s}^{\prime }$ [Eq. (3)] and $\mu_{a}$ [Eq. (4)]. The constraining equation for $\mu_{s}^{\prime }$ is

$$\mu_{s}^{\prime }(\lambda )=\mu_{s}^{\prime }(\lambda_{0})\times {\left[\frac{\lambda }{\lambda_{0}}\right]}^{-B},$$ (3)

where $\mu_{s}^{\prime }(\lambda )$ is the reduced scattering coefficient, $\mu_{s}^{\prime }(\lambda_{0})$ is the reduced scattering coefficient at a reference of 630 nm, λ is all wavelengths, $\lambda_{0}$ is the 630 nm, and B is the scattering exponent, which relates to the size of tissue scatterers; smaller values of B correspond to larger scatterer sizes.^18^^,^^29^ Zonios and Dimou^29^ described an in-depth method to calculate spherical scatterer diameter based on B, which can range between 0.2 and 4.0 in tissue. On the other hand, the constraining equation for $\mu_{a}$ is

$$\mu_{a}(\lambda )=C\times \mu_{a,\text{stock}}(\lambda ),$$ (4)

where $\mu_{a}(\lambda )$ is the absorption coefficient, $\mu_{a,\text{stock}}(\lambda )$ is the absorption coefficient of the bovine-Hb stock solution, and C is the volume fraction of bovine-Hb stock solution in the phantom. The $\mu_{a}$ of the bovine-Hb stock solution was determined via a spectrophotometer and the Beer–Lambert law. These constraining equations required initial and boundary conditions, listed in Table 1. Bounds for $\mu_{s}^{\prime }$ at 630 nm were set based on the $\mu_{s}^{\prime }$ limits of the calibration phantoms used to create the LUTs. Bounds for B were set to exceed values commonly observed (∼0.9 to 1.2) in tissue.^30^ Bounds for C were set to be the minimum and maximum values for volume fraction.^31^ Initial conditions did not affect outcomes as long as they were between the lower and upper bounds. Initial and boundary conditions were constant for all validation phantoms and SDSs.

**Table 1:** Boundary conditions for quantifying optical properties of validation phantoms.

Variable	Lower bound	Upper bound
$\mu_{s}^{\prime }$ ($\lambda_{0}$) (${\mathrm{cm}}^{-1}$)	2.0	15.0
B	0.0	4.0
C (%)	0.0	100

**Fig. 5:**
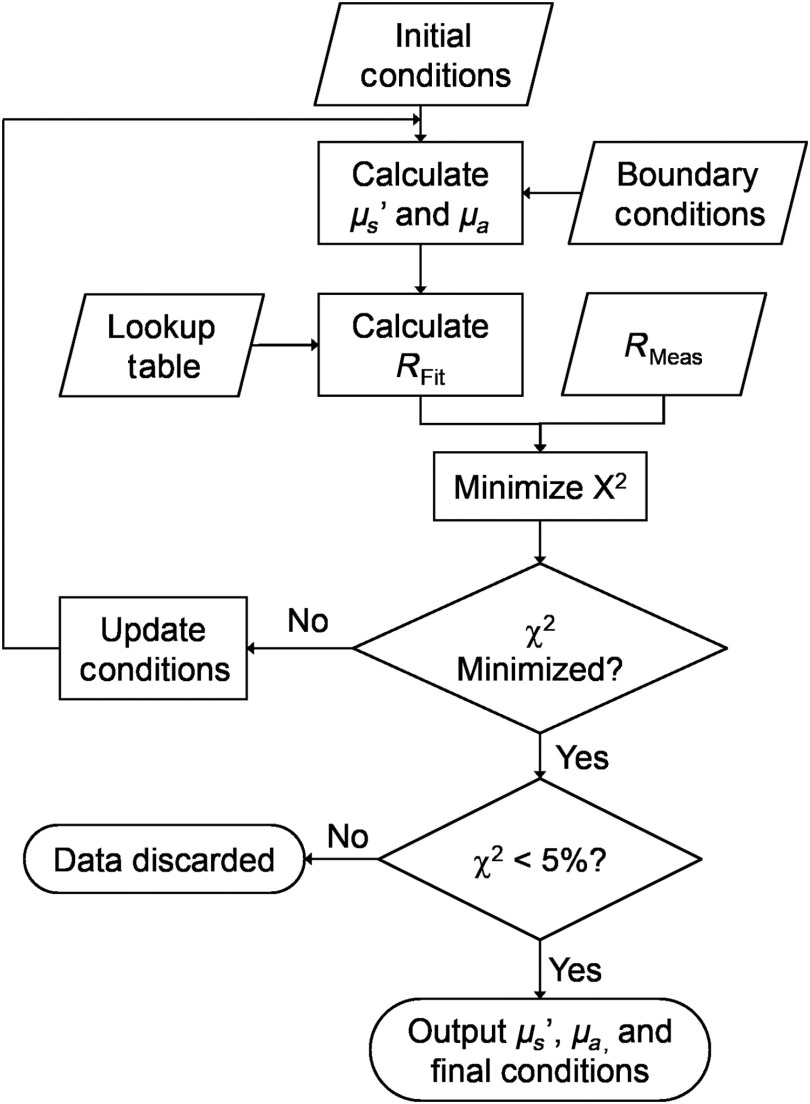
The LUT inverse model of diffuse reflectance fit is based on the damped least-squares nonlinear fitting method, with the goal of outputting $\mu_{s}^{\prime }$ and $\mu_{a}$, as well as contributing parameters from the constraining equations such as scattering exponent and absorber concentration.

After initial conditions were set, the LUT inverse model fit performed up to $1\times 10^{4}$ iterations until the sum of squares ($\chi^{2}$) were minimized^21^ between the fitted reflectance and measured reflectance. All phantom DRS spectra underwent a final quality control step. If $\chi^{2}$ was >5%, data were discarded (Fig. 5).

Percent errors for $\mu_{s}^{\prime }$ and $\mu_{a}$ were calculated by comparing the expected optical properties derived from Mie theory and the Beer–Lambert law to experimental optical properties derived from the LUT inverse model fit. Average percent error was then calculated by averaging the percent error at each wavelength (450 to 900 nm) for each validation phantom (nine phantoms). Percent errors were always positive values; thus, overestimating and underestimating optical properties produced positive errors that did not cancel out. The LUT was considered accurate when average percent errors for $\mu_{s}^{\prime }$ and $\mu_{a}$ were each <10%, a standard cutoff across the literature.^18^^,^^20^^,^^32^^–^^35^

### 2.7 Optical Properties from Balb/c-CT26 Tumor Allografts

After validation of the LUTs, spectra were collected from Balb/c-CT26 tumor allografts $200\pm 50\;{\mathrm{mm}}^{3}$ in diameter (n=9), as well as immediately adjacent tissue from the same mouse. Mice were not anesthetized during data collection. The DRS probe was placed in direct contact with the tissue. Broadband DRS data (450 to 900 nm) were recorded at each SDS (0.75, 2.00, 3.00, and 4.00 mm) with integration times of 100, 200, 300, and 400 ms, respectively, to yield an SNR of at least 15 dB. Five spectra were averaged for all measurements. Spectra were converted to absolute diffuse reflectance values by calibrating with a Spectralon® 20% diffuse reflectance standard and background noise subtraction as previously described.

The optical properties were quantified in a similar manner to validation phantoms, using the LUT inverse model fit based on the damped least squares nonlinear fitting method.^36^ Quantifying *in vivo* $\mu_{s}^{\prime }$ relied on the same constraining equation as validation phantoms [Eq. (3)]. Next, assuming hemoglobin as the only *in-vivo* absorber from 450 to 900 nm, the constraining equation [Eq. (5)] for $\mu_{a}$ was equated as

$$\mu_{a,i}(\lambda )=\mathrm{THC}\times [\alpha \varepsilon_{{\mathrm{HbO}}_{2}}(\lambda )+(1-\alpha )\varepsilon_{Hb}(\lambda )],$$ (5)

where $\mu_{a,i}$ is the initial tissue absorption coefficient, THC is the total hemoglobin concentration in tissue, α is the tissue oxygen saturation (${\mathrm{StO}}_{2}$), and $\varepsilon_{{\mathrm{HbO}}_{2}}(\lambda )$ and $\varepsilon_{Hb}(\lambda )$ are the extinction coefficients of oxyhemoglobin and deoxyhemoglobin, respectively. Next, the final version of the constraining equation for $\mu_{a}$ [Eq. (6)] incorporated the standard pigment-packaging correction factor, described in depth by Rajaram et al.^37^ The corrected absorption equation is equated as

$$\mu_{a,f}(\lambda )=\mu_{a,i}(\lambda )\times [\frac{1-e^{-2\cdot \mu_{a,\mathrm{bl}}(\lambda )\cdot r_{\mathrm{vess}}}}{2\cdot \mu_{a,\mathrm{bl}}(\lambda )\cdot r_{\mathrm{vess}}}],$$ (6)

where $\mu_{a,f}$ is the final tissue absorption coefficient, $\mu_{a,i}$ is the initial absorption coefficient, $\mu_{a,\mathrm{bl}}$ is the absorption coefficient of whole blood assuming a hemoglobin concentration of 150 mg/mL, and $r_{\text{vess}}$ is the average blood vessel radius in the sampled tissue. Including the pigment-packaging correction factor accounts for hemoglobin in tissue being confined to blood vessels, which is a small fraction of the total volume sampled by light. This phenomenon differs from the homogenous tissue-simulating phantoms; however, like the homogenous tissue-simulating phantoms, the constraining equations for *in*-*vivo* measurements of Balb/c-CT26 allografts required initial and boundary conditions, listed in Table 2. Bounds for $\mu_{s}^{\prime }$ at 630 nm were set based on the $\mu_{s}^{\prime }$ limits of the calibration phantoms used to create the LUTs. Bounds for B were set to exceed values commonly observed (∼0.9 to 1.2) in tissue.^30^ Bounds for THC were set such that the maximum could not exceed the hemoglobin concentration found in whole blood (150  mg/mL). Bounds for ${\mathrm{StO}}_{2}$ were set such that the maximum could not exceed the ${\mathrm{StO}}_{2}$ found in fully oxygenated tissue (100%). For $r_{\text{vess}}$, average capillary radius is ∼2.5  μm,^38^ whereas average arteriole radius is ∼10 to 15  μm.^39^ Bounds for $r_{\text{vess}}$ were set to significantly exceed these averages. Initial conditions (Fig. 5) did not affect outcomes as long as they were between the lower and upper bounds. Initial and boundary conditions were constant for all *in*-*vivo* measurements and for all SDSs.

**Table 2:** Boundary conditions for quantifying optical properties of Balb/c-CT26 tissue.

Variable	Lower bound	Upper bound
$\mu_{s}^{\prime }$ ($\lambda_{0}$) (${\mathrm{cm}}^{-1}$)	2.0	15.0
B	0.0	4.0
THC (mg/mL)	0	150
${\mathrm{StO}}_{2}$ (%)	0.0	100
$r_{\text{vess}}$ (μm)	0	100

After initial conditions were set, the LUT inverse model fit performed up to $1\times 10^{4}$ iterations until the sum of squares ($\chi^{2}$) were minimized between the fitted reflectance and measured reflectance. Using the constraining equations for *in*-*vivo* tissue, $\mu_{s}^{\prime }$ at 630 nm, THC, and ${\mathrm{StO}}_{2}$ were quantified as functions of tissue types (normal versus tumor) and SDS. Optical properties were compared between normal and tumor tissues for each SDS. The significance threshold was set at 0.05. All *in*-*vivo* DRS spectra underwent a final quality control step. If $\chi^{2}$ was >5%, data were discarded (Fig. 5). Artifacts due to mouse movement during data collection could potentially cause a high $\chi^{2}$ between the fitted reflectance and measured reflectance. Significance of optical properties between tissue types (healthy and tumor) and SDS (0.75, 2.00, 3.00, and 4.00) was determined using a two-way mixed ANOVA. The significance level was set at 0.05.

### 2.8 Sampling Depth of Diffuse Reflectance Spectroscopy Probe into Tissue

The next goal was to quantify sampling depth for each SDS as a function of $\mu_{s}^{\prime }$ and $\mu_{a}$. In other words, once $\mu_{s}^{\prime }$ and $\mu_{a}$ have been quantified via the LUT inverse model, at what depth into tissue are these optical properties being measured?

**Fig. 6:**
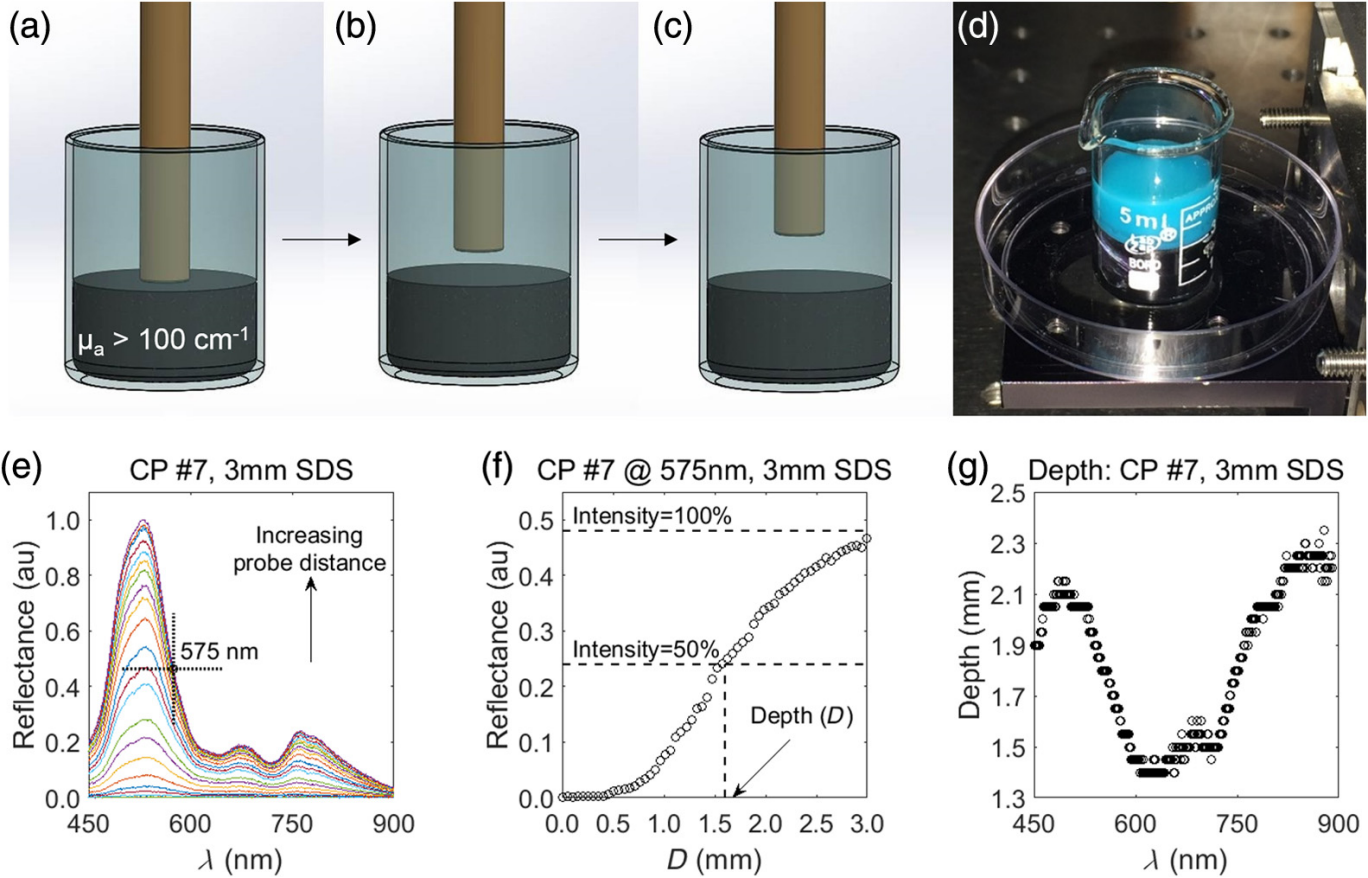
Sampling depth was quantified by (a–d) taking DRS measurements of calibration phantoms at 50-μm increments between 0 and 3 mm from a highly absorbing ($\mu_{a}>100\;{\mathrm{cm}}^{-1}$) phantom layer. (e) Reflectance (R) increased as distance between the probe and black layer increased, shown for calibration phantom #7 at the 3.00-mm SDS as an example. (f) Sampling depth (D) was defined when the SDS is most sensitive to the black layer, which occurs when 50% of photons reach the black layer. (g) Sampling depth (D) was then quantified at the 50-μm increment at each wavelength.

To quantify sampling depth, a 5×3 (15 total) set of calibration phantoms were constructed.^18^ Each of these phantoms was placed into a 5-mL beaker (Fig. 6) with a highly absorbing ($\mu_{a}>100\;{\mathrm{cm}}^{-1}$) black phantom layer, made with (poly)-dimethylsiloxane (PDMS) and black India ink at the bottom. It was assumed that any photon contacting this layer would be attenuated. The $\mu_{a}$ of the black layer was calculated using a spectrophotometer and the Beer–Lambert Law. Additionally, the black layer contained no scattering agent. Contributions from specular reflection at the interface between the black layer and calibration phantoms were negligible (data not shown) as there is a minimal mismatch between the PDMS and liquid phantoms.^14^^,^^18^

The probe was placed in direct contact with the black layer [Figs. 6(a)–6(d)]. Using a mechanical translation stage equipped with a micrometer scale (LNR25M, Thorlabs), the probe was raised from the black layer in 50-μm increments from 0 to 3 mm. DRS measurements, from 450 to 900 nm, were taken at each 50-μm step at each SDS of 0.75, 2.00, 3.00, and 4.00 mm at integration times of 100, 200, 300, and 400 ms, respectively, to yield an SNR of at least 15 dB. As the optical properties of the calibration phantoms were known, a relationship was established between $\mu_{s}^{\prime },\mu_{a}$, and reflectance at various sampling depths. As the probe increased in distance from the black layer, reflectance increased, then leveled [Fig. 6(e)]. At each wavelength, the probe was most sensitive to changes in optical properties when 50% of photons reached the black layer [Fig. 6(f)]. When this process was repeated at each wavelength, a relationship between sampling depth and wavelength was established [Fig. 6(g)]. Therefore, sampling depth (λ) was defined at the most sensitive 50-μm increment.

Three-dimensional (3-D) plots were generated for each SDS by plotting sampling depth (D) against $\mu_{s}^{\prime }$ and $\mu_{a}$ and then interpolating between raw data points to create a smooth mesh for $\mu_{s}^{\prime }$ between 4 and $12\;{\mathrm{cm}}^{-1}$, and $\mu_{a}$ between 0 and $8\;{\mathrm{cm}}^{-1}$. This optical property range accounts for all expected $\mu_{s}^{\prime }$ and $\mu_{a}$ in murine tissue in the wavelength range of interest (450 to 900 nm). Once optical properties were calculated using the LUT inverse model, sampling depth was quantified. Significance of sampling depth between tissue types (healthy and tumor) and SDS (0.75, 2.00, 3.00, and 4.00) was determined using a two-way mixed ANOVA. The significance level was set at 0.05.

## 3 Results

### 3.1 Tumor Allograft Geometry by Tissue Type

**Fig. 7:**
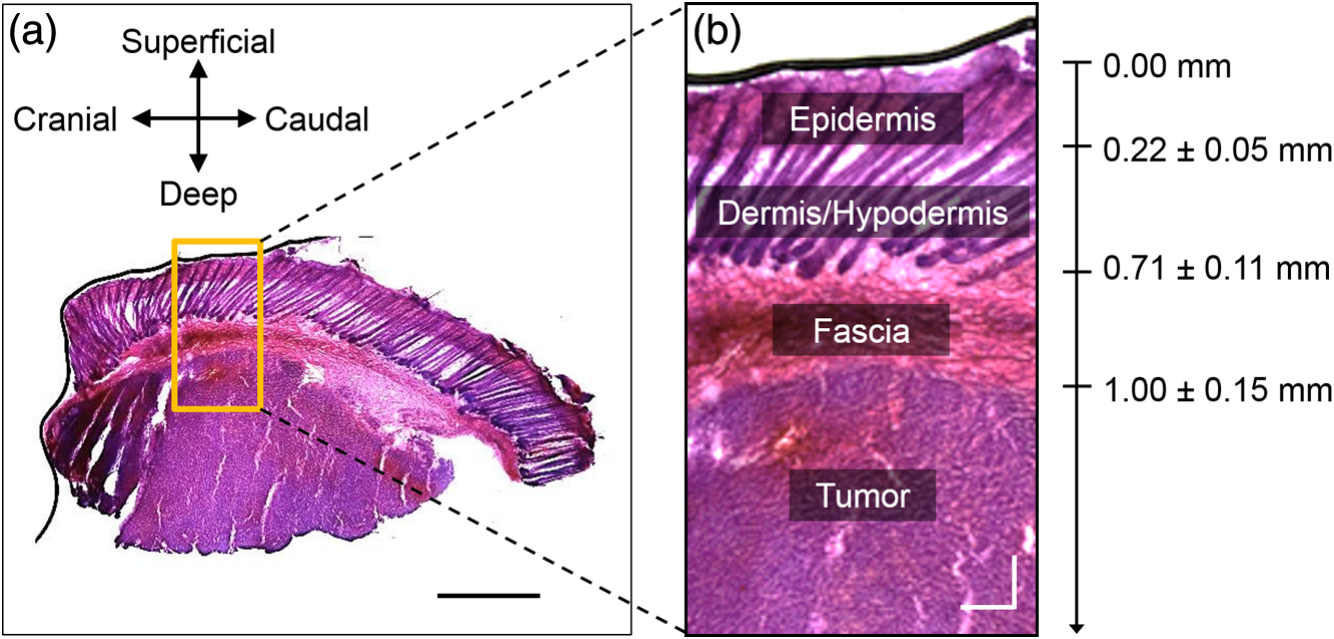
To acquire optical properties from the subcutaneous tumor, broadband light from the DRS probe needed to penetrate past the fascia, located 1.00±0.15  mm from the surface. Values are mean ± SD. (scale bar = 1 mm).

Tumors were dissected, cut into 6-μm sections, and H&E stained. Three primary tissue types were visualized above the subcutaneous tumor (Fig. 7): the epidermis, dermis/hypodermis, and fascia. In female Balb/c-CT26 tumor allografts (n=9), the epidermis was 0.22-±0.05-mm thick, the base of the dermis was 0.71±0.11  mm from the surface, and the base of the fascia was 1.00±0.15  mm from the surface, respectively.

### 3.2 Validation of Lookup-Table Inverse Model

**Fig. 8:**
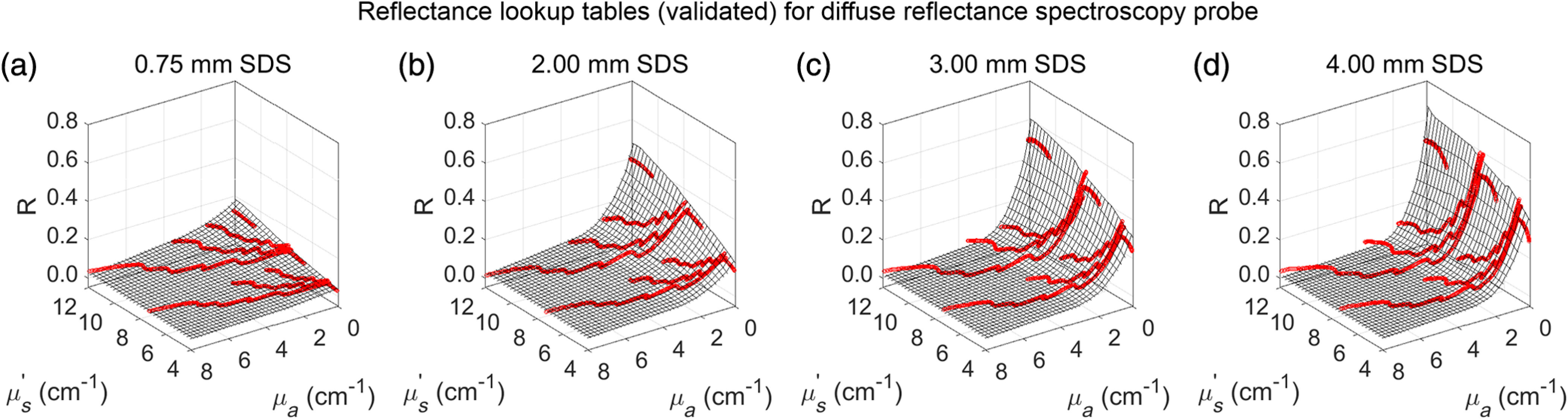
Reflectance from bovine Hb-based validation phantoms (red) was plotted against the LUTs (grayscale grid) for each SDS of (a) 0.75, (b) 2.00, (c) 3.00, and (d) 4.00 mm.

The reflectance from each validation phantom at each SDS, with known $\mu_{s}^{\prime }$ and $\mu_{a}$, was plotted against the LUT created from the calibration phantoms (Fig. 8). Percent errors were acceptable if <10% for both $\mu_{s}^{\prime }$ and $\mu_{a}$.

**Fig. 9:**
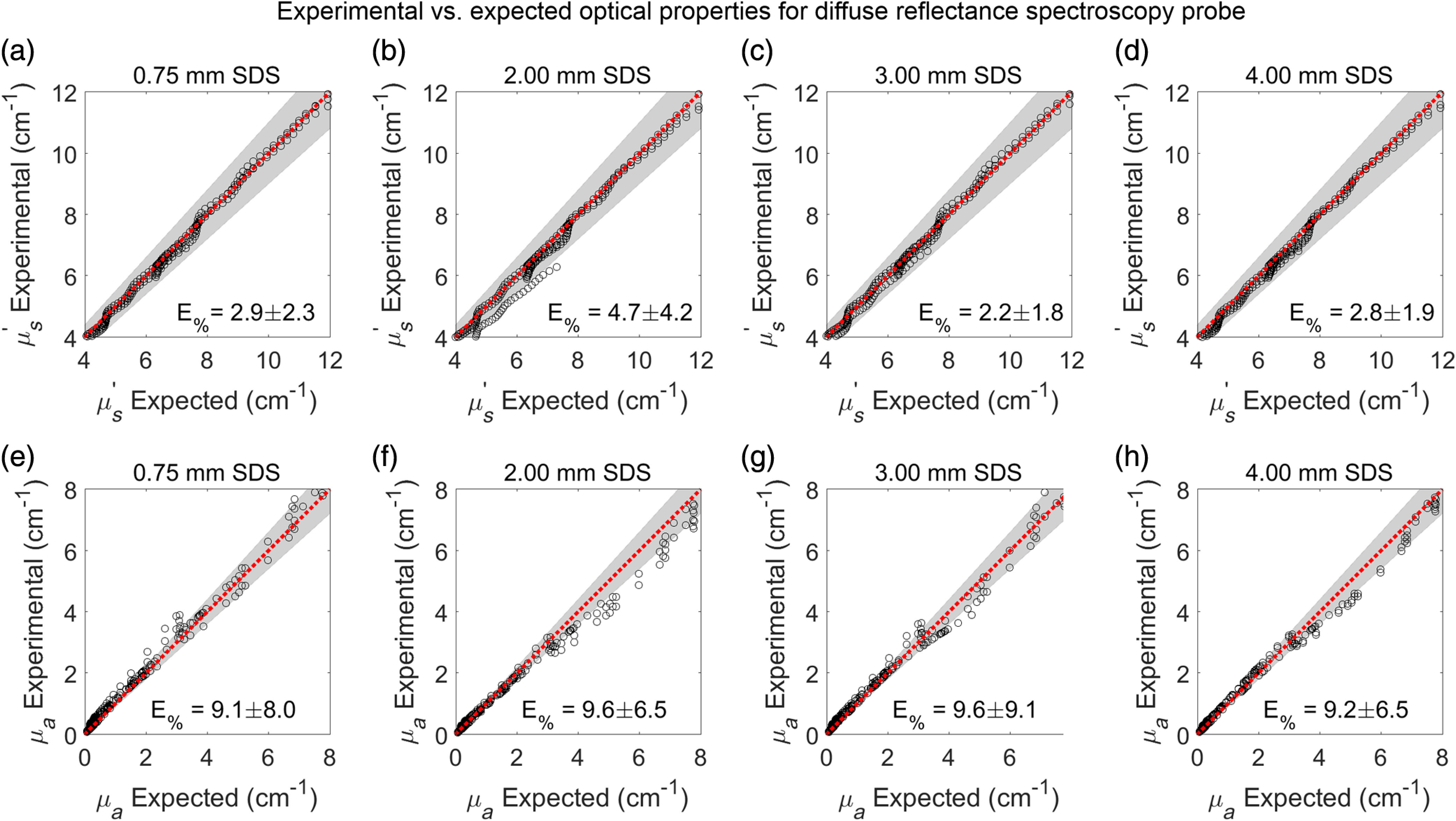
Percent errors for comparing experimental optical properties (from LUT inverse model fit) and expected (known) optical properties were below 10% for all SDS of (a, e) 0.75, (b, f) 2.00, (c, g) 3.00, and (d, h) 4.00 mm. The LUT inverse model fit more accurately extracted (a-d) $\mu_{s}^{\prime }$ (percent errors <5%) compared WITH (e–h) $\mu_{a}$. Black dots represent raw data. Red lines indicate a perfect fit with 0% error. Gray background represents the acceptable 10% error.

Average percent errors for $\mu_{s}^{\prime }$ were 2.9%, 4.7%, 2.2%, and 2.8% for the 0.75-, 2.00-, 3.00-, and 4.00-mm SDSs, respectively. Average percent errors for $\mu_{a}$ were 9.1%, 9.6%, 9.6%, and 9.2% for the 0.75-, 2.00-, 3.00-, and 4.00-mm SDSs, respectively. Thus, all percent errors were below 10% (Fig. 9).

### 3.3 Sampling Depth in Balb/c-CT26 Allografts

**Fig. 10:**
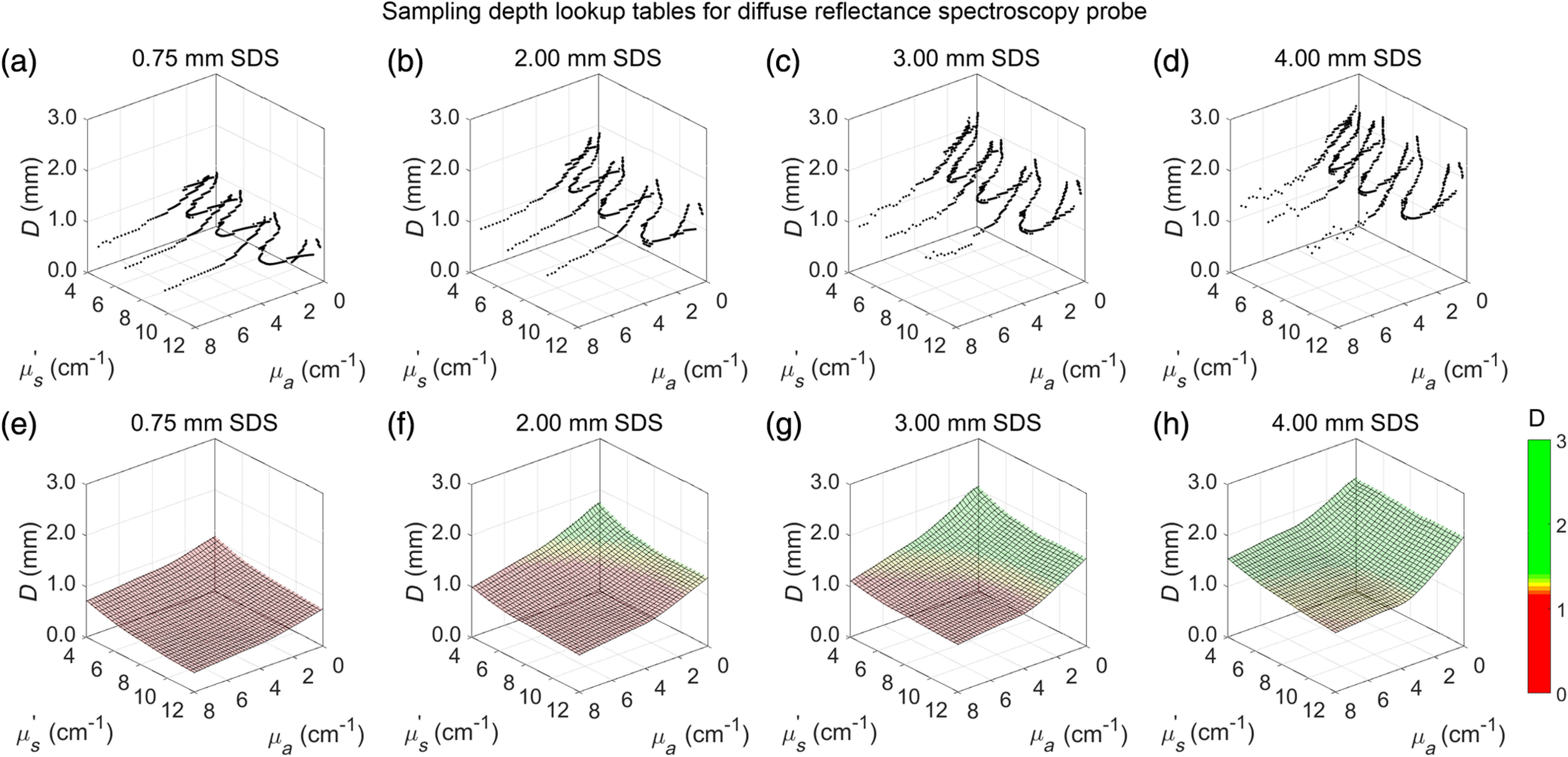
Raw sampling depth data (a–d) was plotted for each SDS and then (e–h) interpolated into a mesh. Sampling depth increased as SDS increased.

Following DRS measurements of calibration phantoms overlying a highly absorbing PDMS layer, 3-D plots were generated for each SDS by plotting sampling depth (D) against $\mu_{s}^{\prime }$ and $\mu_{a}$ and then interpolating between raw data points to create a smooth mesh. Sampling depths were valid for $\mu_{s}^{\prime }$ between 4 and $12\;{\mathrm{cm}}^{-1}$, and $\mu_{a}$ between 0 and $8\;{\mathrm{cm}}^{-1}$ (Fig. 10). Lowest sampling depth occurred at the highest optical properties ($\mu_{s}^{\prime }=12\;{\mathrm{cm}}^{-1}$, $\mu_{a}=8\;{\mathrm{cm}}^{-1}$) and highest sampling depth occurred at the lowest optical properties ($\mu_{s}^{\prime }=4\;{\mathrm{cm}}^{-1}$, $\mu_{a}=0\;{\mathrm{cm}}^{-1}$). Based on this, sampling depths ranged between 0.37 and 1.10 mm, 0.72 and 1.76 mm, 0.92 and 2.08 mm, and 1.16 and 2.25 mm for the 0.75-, 2.00-, 3.00-, and 4.00-mm SDSs, respectively, indicating sampling depth increased as SDS increased. Subcutaneous tumors were located 1.00±0.15  mm or deeper below the skin surface; thus, broadband light from the DRS probe needed to penetrate at least 1.15 mm into tissue to sample tumor optical properties. With regard to the colormap in Fig. 10, red coloring indicates sampling depth ≤1.15  mm, which was the average thickness, plus one standard deviation, of the overlying skin and fascia of the subcutaneous Balb/c-CT26 tumor. Yellow coloring indicates sampling depth between 1.15 and 1.45 mm, with peak yellow occurring at 1.30 mm, which was the average thickness, plus two standard deviations. Green coloring indicates sampling depth ≥1.45  mm, which was the average thickness plus three standard deviations. Thus, yellow and green coloring represent optical properties in which tumor tissue was sampled.

### 3.4 Balb/c-CT26 Allograft Wavelength-Dependent Optical Properties

**Fig. 11:**
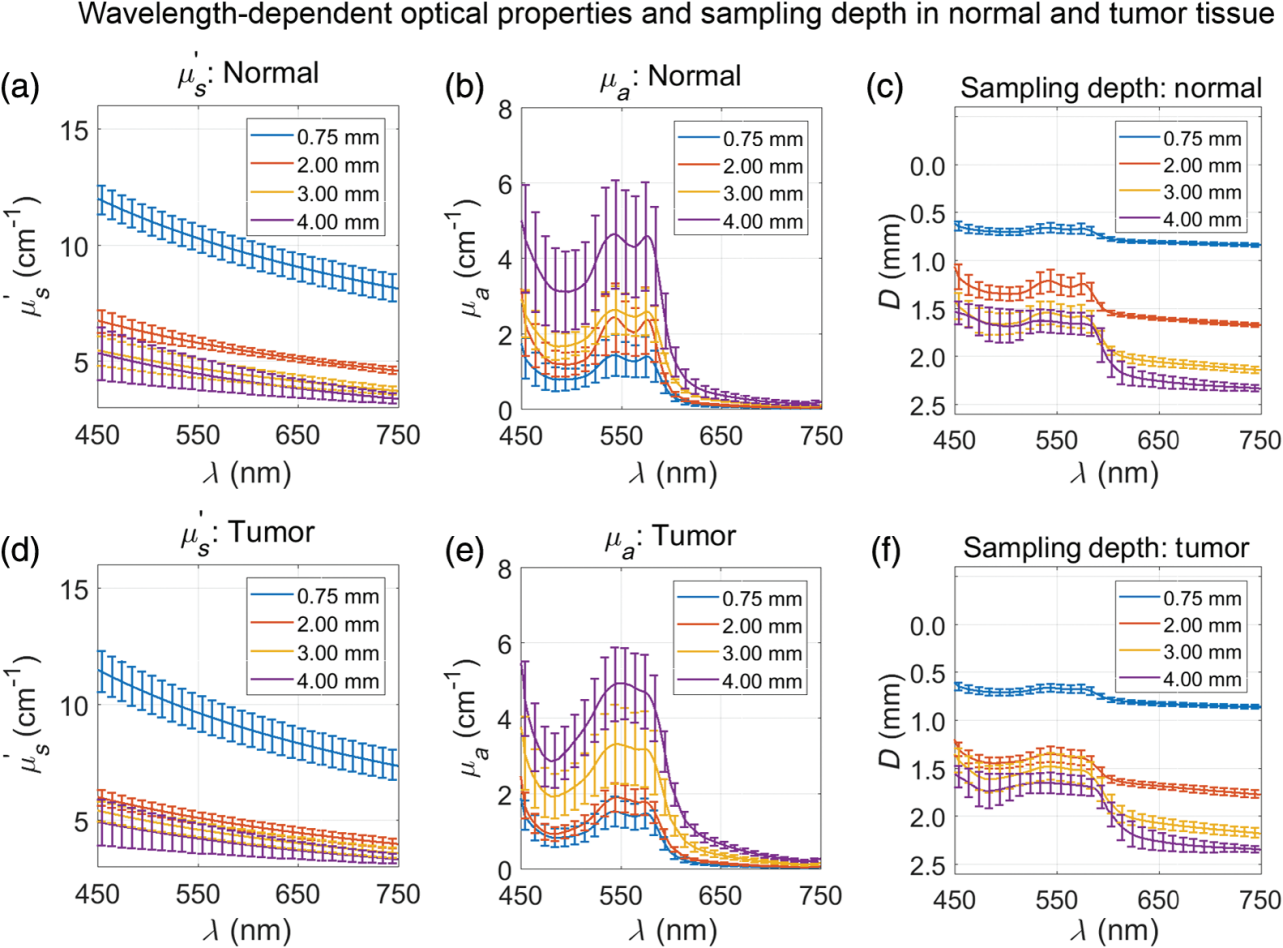
Average optical properties and sampling depth for (a–c) normal Balb/c flank tissue and (d–f) subcutaneous Balb/c-CT26 tumor allografts showing (a, d) $\mu_{s}^{\prime }$, (b, e) $\mu_{a}$, and (c, f) sampling depth. As SDS increased, $\mu_{s}^{\prime }$ decreased, $\mu_{a}$ increased, and sampling depth increased for both tissue types. Values are mean ± SD.

Next, DRS measurements were collected from Balb/c-CT26 tumor allografts (n=9) $200\pm 50\;{\mathrm{mm}}^{3}$ in diameter, as well as immediately adjacent normal flank tissue from the same mouse. The LUT inverse model fit analyzed the spectra to output $\mu_{s}^{\prime }(\lambda )$ and $\mu_{a}(\lambda )$ (Fig. 11). Based on the relationship between $\mu_{s}^{\prime }$, $\mu_{a}$, and sampling depth (Fig. 10), sampling depth was quantified as a function of wavelength. In general, as SDS increased, $\mu_{s}^{\prime }(\lambda )$ decreased, $\mu_{a}(\lambda )$ increased, and sampling depth increased for both normal and tumor tissues.

### 3.5 Balb/c-CT26 Allograft Diffuse Reflectance Spectroscopy-Derived Physiological Parameters

**Fig. 12:**
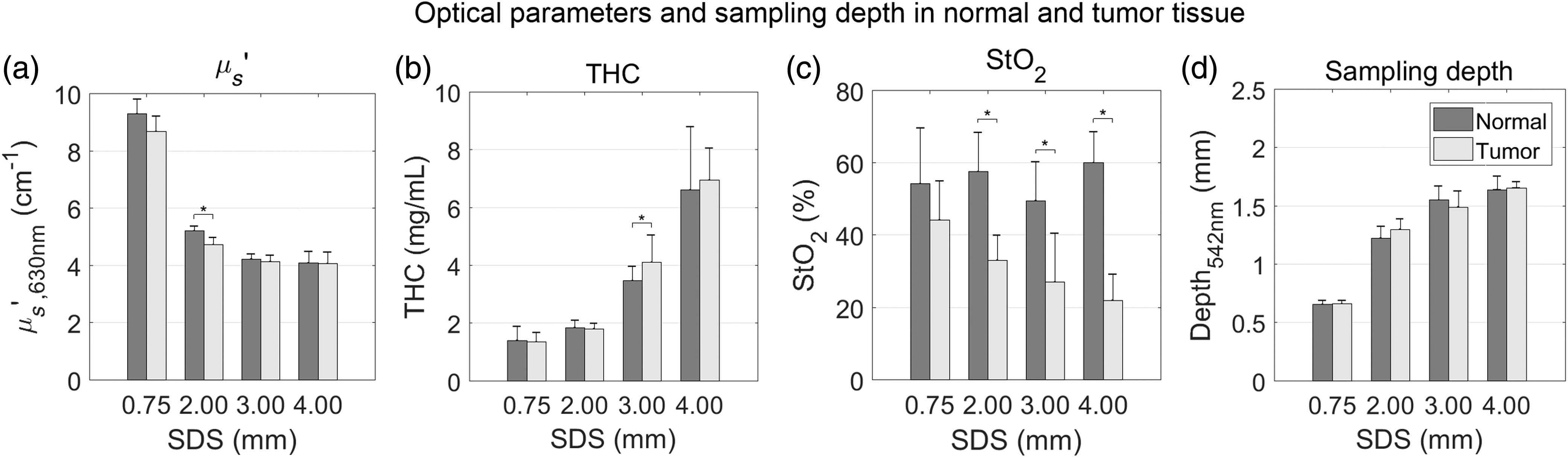
Average (a) $\mu_{s}^{\prime }$ at 630 nm, (b) THC, (c), ${\mathrm{StO}}_{2}$, and (d) sampling depth for normal (dark gray) and tumor (light gray) tissue. The $\mu_{s}^{\prime }$ was comparable between normal and tumor tissues and decreased as SDS increased. The THC was comparable between normal and tumor tissues and increased as SDS increased. The ${\mathrm{StO}}_{2}$ in tumor tissue was significantly decreased compared with normal tissue for SDSs longer than 0.75 mm. Additionally, ${\mathrm{StO}}_{2}$ decreased as SDS decreased. The sampling depth was comparable between normal and tumor tissues and increased as SDS increased. Values are mean ± SD. Significance is indicated by ^*^(p<0.05).

After comparing wavelength-dependent optical properties as a function of SDS, key physiological optical parameters were extracted and compared for normal and tumor tissues (Fig. 12).

The $\mu_{s}^{\prime }$ at 630 nm decreased as SDS increased [Fig. 12(a)], as shown in Fig. 11. For the 0.75-, 3.00-, and 4.00-mm SDSs, differences in $\mu_{s}^{\prime }$ were insignificant between normal and tumor tissues. In the 2.00-mm SDS, $\mu_{s}^{\prime }$ was significantly lower in tumor tissue (p=0.02) compared with normal tissue but only by $0.49\;{\mathrm{cm}}^{-1}$.

The THC, measured in mg/mL, increased as SDS increased [Fig. 12(b)]. This was also indicated by the observed increased $\mu_{a}(\lambda )$ magnitude shown in Fig. 11. For the 0.75-, 2.00-, and 4.00-mm SDSs, differences in THC were insignificant between normal and tumor tissues. In the 3.00-mm SDS, THC was significantly lower in normal tissue (p=0.03) compared with tumor tissue but only by 0.64  mg/mL. The THC rose from ∼1.4  mg/mL in the 0.75-mm SDS to ∼6.8  mg/mL in the 4.00-mm SDS for both tissue types.

The ${\mathrm{StO}}_{2}$ remained constant as SDS increased in normal tissue [Fig. 12(c)]; however, ${\mathrm{StO}}_{2}$ decreased as SDS increased in tumor tissue, indicating increasing hypoxia at increased depths within the tumor microenvironment. The ${\mathrm{StO}}_{2}$ quantified by the short 0.75-mm SDS was significantly higher (p<0.01) than the ${\mathrm{StO}}_{2}$ quantified by all longer SDSs. Furthermore, there was no significant difference in ${\mathrm{StO}}_{2}$ between normal and tumor tissues in the 0.75-mm SDS. However, tumor tissue expressed significantly decreased ${\mathrm{StO}}_{2}$ compared with normal tissue for SDSs of 2.00, 3.00, and 4.00 mm.

In Fig. 12(d), sampling depth was quantified at the first Q-band of hemoglobin at 542 nm, where the lowest sampling depth would occur. In normal tissue, sampling depth was 0.66±0.04, 1.22±0.11, 1.55±0.12, and 1.64±0.12  mm at 542 nm for the 0.75-, 2.00-, 3.00-, and 4.00-mm SDSs, respectively. In tumor tissue, sampling depth was 0.66±0.03, 1.30±0.09, 1.49±0.14, and 1.65±0.05  mm at 542 nm for the 0.75-, 2.00-, 3.00-, and 4.00-mm SDSs, respectively. There was no significant difference (p>0.05) comparing sampling depth in normal versus tumor tissue. For both normal and tumor tissues, sampling depth increased significantly (p<0.01) at longer SDSs of 2.00, 3.00, and 4.00 mm compared with the shorter 0.75-mm SDS.

## 4 Discussion

A DRS probe was designed to acquire optical properties of subcutaneous murine tumor allografts and was applied specifically in Balb/c-CT26 colon tumor allografts. In this paper, a complete validation of the DRS probe in this context was presented. Raw data from DRS are reflectance intensities as functions of wavelength. This paper explicitly describes a method to postprocess raw spectra into the associated optical properties, $\mu_{s}^{\prime }$ and $\mu_{a}$, physiological perfusion parameters including THC and ${\mathrm{StO}}_{2}$, and sampling depth.^14^^,^^40^ The central hypothesis was that this DRS probe would simultaneously sample the overlying epithelial skin layer as well as the subcutaneous tumor allograft by including multiple discrete SDSs and extract optical parameters from increasing depths.^14^^,^^41^ DRS data at each SDS represent a weighted average of physiological parameters collected from increasing sampling depths. In the female Balb/c-CT26 colon tumor allograft model, the skin, consisting of the epidermis, dermis, and hypodermis was 0.71-±0.11-mm thick, and the underlying fascia resulted in 1.00±0.15  mm of total tissue above the underlying subcutaneous tumor. These values are expected to vary based on mouse strain, subcutaneous tumor location, sex, and age, and should be independently confirmed by investigators performing similar studies.^27^^,^^42^^,^^43^ Thus, the DRS probe needed to sufficiently sample beyond the 1.00-mm skin layer and into the subcutaneous tumor.

An LUT-based inverse model, an established method, was chosen as the method to relate diffuse reflectance with $\mu_{s}^{\prime }$ and $\mu_{a}$.^18^^,^^19^^,^^36^^,^^40^ Other methods exist to perform this task such as Monte Carlo-based simulations,^14^^,^^15^^,^^44^ but the LUT-based inverse model was chosen because it is based on experimental values that necessarily account for our specific system response.^36^ To generate an LUT, a set of calibration phantoms with known optical properties was used. As of the current report, the LUTs for each SDS are valid for $\mu_{s}^{\prime }$ between 4 and $12\;{\mathrm{cm}}^{-1}$ at 630 nm and for $\mu_{a}$ between 0 and $8\;{\mathrm{cm}}^{-1}$. This optical property range effectively encompasses expected optical properties found in murine skin and subcutaneous tumors between 450 and 900 nm.^27^ This wavelength range was chosen because of the absorption properties of hemoglobin, with specific absorption peaks (Q-bands) at 542 and 576 nm that indicate THC and ${\mathrm{StO}}_{2}$ and negligible absorption ($\mu_{a}<0.5\;{\mathrm{cm}}^{-1}$ for both oxygenated and deoxygenated hemoglobin in whole blood) between 600 and 900 nm.^45^ Therefore, reflectance in the 600- to 900-nm wavelength range necessarily indicates $\mu_{s}^{\prime }$.^21^ It is common in the literature to report $\mu_{s}^{\prime }$ at a reference of 630 nm, so this convention was used here.^21^^,^^37^^,^^46^^,^^47^

As the LUTs were generated with dye-based calibration phantoms, a set of bovine hemoglobin-based phantoms, which more closely simulate physiological conditions, with known optical properties was used to validate the LUTs.^18^ Within the LUT optical property range, it was shown that average percent errors for extracting $\mu_{s}^{\prime }$ and $\mu_{a}$ were below 10% for all SDSs, indicating it is reasonable to assume that measured tissue optical properties and physiological perfusion metrics are accurate within 10%. Average percent errors below 10% are considered acceptable and are comparable with many other DRS studies.^32^^–^^35^ However, it was not uncommon for percent errors in several experimental optical property observations to exceed 10% (Fig. 9). Despite this, there was no relationship between percent error and magnitude of $\mu_{s}^{\prime }$ and $\mu_{a}$, indicating that while some *in-vivo* measurements of murine tissue may indeed exceed 10%, on average the percent errors will be within 10% regardless of magnitude of $\mu_{s}^{\prime }$ and $\mu_{a}$. Additionally, percent errors did not significantly change with respect to SDS. As SDS is related to sampling depth,^14^ this indicates that measuring optical properties of deeper tumor tissue were no less accurate than measuring optical properties of shallower skin tissue. It should be noted that average percent errors for extracting $\mu_{a}$ were > (∼9%) compared with extracting $\mu_{s}^{\prime }$ (∼3%), a common observation in existing literature.^46^ Finally, as LUT validation was performed with bovine hemoglobin-based phantoms, it was extraneous to perform additional validation via Monte Carlo simulations.

Once the relationship between diffuse reflectance, $\mu_{s}^{\prime }$, and $\mu_{a}$ was established and validated, the same set of calibration phantoms was used to establish a relationship between sampling depth, $\mu_{s}^{\prime }$, and $\mu_{a}$. As of the current report, the sampling depth relationship for each SDS are valid for $\mu_{s}^{\prime }$ between 4 and $12\;{\mathrm{cm}}^{-1}$ at 630 nm and for $\mu_{a}$ between 0 and $8\;{\mathrm{cm}}^{-1}$. We employed a method to quantify sampling depth similar to that pioneered by Hennessey et al.^14^ in which sampling depth as a function of wavelength was quantified based on the depth at which the SDS was most sensitive to an optical heterogeneity (Fig. 6). It is important to note that, even at lower and higher depths, the probe was still sensitive to the optical heterogeneity [Fig. 6(f)], similar to other studies.^14^ This shows that stating the probe has a single sampling depth at specific optical properties is an oversimplification. Instead, the depth sampled by our DRS probe represents a wide range, a phenomenon described explicitly by Kanick et al.^13^ However, for simplicity, we report sampling depth as a single value at which the SDS was most sensitive to the heterogeneity. Figure 10 shows that sampling depth increased with increasing SDS and decreased with increasing $\mu_{s}^{\prime }$ and $\mu_{a}$, as expected.^13^^,^^14^

There was a decrease in sampling depth at the Soret band (<500  nm) and Q-bands (542 and 576 nm) of hemoglobin. It is these peaks that most heavily influence extracted THC and ${\mathrm{StO}}_{2}$ in the LUT inverse model. Thus, even though sampling depth is higher at longer wavelengths, we explicitly report sampling depth at the first Q-band of hemoglobin, where sampling depth is lowest in our wavelength range (450 to 900 nm). From Fig. 12(d), we can conclude that the 0.75-mm SDS only samples the skin layer as its sampling depth was 0.66±0.04  mm and 0.66±0.03  mm for normal and tumor tissues, respectively, shallower than the 1.00-±0.15-mm normal tissue above the subcutaneous tumor. Further evidence for the 0.75-mm SDS sampling only the overlying skin layer is shown from Figs. 12(a)–12(c), in which there were insignificant differences between normal versus tumor tissues with respect to $\mu_{s}^{\prime }$, THC, and ${\mathrm{StO}}_{2}$. The tumor begins to be sampled at the 2.00-, 3.00-, and 4.00-mm SDSs, indicated by the sampling depths at the first Q-band to be 1.30±0.09, 1.49±0.14, and 1.65±0.05  mm, respectively. As such, as the subcutaneous tumor is relatively hypoxic,^48^ there was a significant difference in ${\mathrm{StO}}_{2}$ between normal and tumor tissues at these sampling depths [Fig. 12(c)]. Furthermore, in tumor tissue, ${\mathrm{StO}}_{2}$ decreased steadily from 44%±11% to 22%±7% as SDS increased. It is important to note that the observed decreasing ${\mathrm{StO}}_{2}$ with increasing sampling depth does not necessarily indicate the tumor was more hypoxic at increased depths but is most likely due to more overall tumor tissue contributing to the volume-averaged physiological parameters. It is common for DRS-derived ${\mathrm{StO}}_{2}$ of keratinized epithelia, such as the skin, to be much <100%.^10^ DRS studies reporting in nonkeratinized epithelia tend to extract much higher ${\mathrm{StO}}_{2}$ values upward of 95%.^18^ Additionally, the ${\mathrm{StO}}_{2}$ presented in this study does not necessarily correlate with arterial oxygen saturation, which would be more accurately measured using pulse oximetry.^49^ Interestingly, $\mu_{s}^{\prime }$ and THC were mostly comparable between normal and tumor tissues, indicating ${\mathrm{StO}}_{2}$ may be a key physiological parameter to evaluate murine tissue health via DRS, a sentiment held by other research groups.^9^^,^^10^^,^^50^

Figures 11 and 12 show that increasing sampling depth resulted in decreased $\mu_{s}^{\prime }$ and increased $\mu_{a}$ in both normal and tumor tissues. In the skin, scattering from the epidermis (0 to 0.22 mm) is primarily caused by keratin, a filamentous protein, as well as cell nuclei and lipid membranes. In the dermis and hypodermis, (0.22 to 0.71 mm) scattering is primarily caused by collagen, which accounts for ∼25% of the dermal volume, other cellular constituents, ^5^^–^^7^ and lipids confined to adipocytes in the hypodermis. In the superficial fascia (0.71 to 1.00 mm), an areolar connective tissue,^5^^,^^51^ scattering is primarily caused by collagenous, elastic, and reticular fibers. Finally, scattering in the CT26 cell layer (an epithelial cell type) is caused by cellular constituents. Measurements in similar tissue in the literature have suggested that epithelial tumors have lower light scattering^52^ compared with skin, whose scattering properties are dominated by the dermis,^7^ although a direct comparison of $\mu_{s}^{\prime }$ between subcutaneous CT26 allografts and skin has not been exclusively studied. The $\mu_{s}^{\prime }$ presented here were comparable with other studies.^27^ On the other hand, increased $\mu_{a}$, associated with increased THC, increased with sampling depth. The epidermis contains no blood vessels, which are situated in deeper dermal layers.^27^ In Balb/c mice, which are albino, hemoglobin is the only significant absorber. It is important to note that the observed increasing THC with increasing sampling depth does not necessarily indicate higher THC in the tumor but is most likely due to reduced contribution of the epidermis to the volume-averaged optical properties of the subcutaneous tumor model. The $\mu_{a}$ and THC presented here were comparable with other studies.^7^^,^^27^^,^^53^^,^^54^

This work has several limitations. First, contact-based, noninvasive DRS cannot sample into the center of a subcutaneous tumor $200\pm 50\;{\mathrm{mm}}^{3}$ in diameter. Sampling into the tumor center may be difficult even for small tumors, since even at the 4.00-mm SDS, sampling depth only reached 1.65±0.05  mm. Therefore, considering the spatial heterogeneity of the tumor microenvironment,^55^ DRS may not provide representative data for the entire tumor. Spliethoff et al.^10^ overcame this limitation using a minimally invasive biopsy needle with integrated optical fibers for intratumoral DRS measurements in subcutaneous murine xenografts. Second, extracted optical properties are volume-averaged, meaning that fine spatial resolution of $\mu_{s}^{\prime }$ and $\mu_{a}$ is lost.^13^^,^^56^ Moreover, even at long SDSs designed to sample deeper into the subcutaneous tumor allograft, extracted optical properties are a volume-averaged measurement of both tumor and skin. To overcome this limitation, the 0.75-mm SDS was integrated into the DRS probe design to simultaneously and exclusively sample overlying skin. This way, fluctuations in optical properties and physiological parameters over time could be attributed to either changes in the tumor itself, or changes in the skin, and assumptions could be limited. Saager et al.^56^ mitigated this volume-average limitation by implementing a depth-resolved quantification based on a two-layer Monte Carlo model. Additionally, due to the thin nature of skin, we expect overall optical contributions on tumor physiological parameters to be relatively small.^57^ Finally, future work must correlate DRS-derived perfusion metrics with immunohistochemical analysis. For example, pimonidazole is a dye that stains for hypoxia^58^ and can be used to correlate end-point hypoxic fraction of tumor sections with *in-vivo* ${\mathrm{StO}}_{2}$ measurements via DRS.

## 5 Conclusion

DRS is a noninvasive spectral biopsy tool that has shown promise in early cancer diagnostics, tracking tissue response to therapy, and in intraoperative surgical guidance. This paper provides an outline for a general method for quantifying tissue optical properties, as well as physiologically relevant perfusion parameters, such as hemoglobin concentration and tissue oxygen saturation, that can be used by investigators hoping to implement DRS for cancer research. Experimental methods presented here are scalable for smaller probe sizes (within the subdiffuse regime) for endoscopically deployable spectroscopy probes, where the diffuse approximation is limited.
